# Aberrantly Activated APOBEC3B Is Associated With Mutant p53-Driven Refractory/Relapsed Diffuse Large B-Cell Lymphoma

**DOI:** 10.3389/fimmu.2022.888250

**Published:** 2022-05-03

**Authors:** Xuzhao Zhang, Zhaoxing Wu, Yuanyuan Hao, Teng Yu, Xian Li, Yun Liang, Jinfan Li, Liansheng Huang, Yang Xu, Xiuzhen Li, Xiaohua Xu, Weiqin Wang, Genbo Xu, Xiaohong Zhang, Qinghua Lv, Yongming Fang, Rongzhen Xu, Wenbin Qian

**Affiliations:** ^1^ Department of Hematology, The Second Affiliated Hospital of Zhejiang University School of Medicine, Hangzhou, China; ^2^ Cancer Institute (Key Laboratory of Cancer Prevention and Intervention, China National Ministry of Education), The Second Affiliated Hospital of Zhejiang University School of Medicine, Hangzhou, China; ^3^ Zhejiang Provincial Key Laboratory for Cancer Molecular Cell Biology, Zhejiang University, Hangzhou, China; ^4^ Department of Pathology, The Second Affiliated Hospital of Zhejiang University School of Medicine, Zhejiang University, Hangzhou, China

**Keywords:** *TP53* mutation, APOBEC3B, DLBCL, refractory, relapse

## Abstract

Tumor protein 53 (*TP53*) mutation predicts an unfavorable prognosis in diffuse large B-cell lymphoma (DLBCL), but the molecular basis for this association remains unclear. In several malignancies, the cytidine deaminase apolipoprotein B mRNA editing enzyme catalytic subunit 3B (APOBEC3B) has been reported to be associated with the *TP53* G/C-to-A/T mutation. Here, we show that the frequency of this mutation was significantly higher in relapsed/refractory (R/R) than in non-R/R DLBCL, which was positively associated with the *APOBEC3B* expression level. APOBEC3B overexpression induced the *TP53* G/C-to-A/T mutation *in vitro*, resulting in a phenotype similar to that of DLBCL specimens. Additionally, APOBEC3B-induced p53 mutants promoted the growth of DLBCL cells and enhanced drug resistance. These results suggest that APOBEC3B is a critical factor in mutant p53-driven R/R DLBCL and is therefore a potential therapeutic target.

## Introduction

Diffuse large B-cell lymphoma (DLBCL) is the most common type of lymphoid malignancy in adults and has a heterogeneous clinical course. The combination immunotherapy regimen of rituximab, cyclophosphamide, doxorubicin, vincristine, and prednisone (R-CHOP)—the current standard of care for DLBCL—has greatly improved the outcome of patients, with durable remission achieved in 50% of cases (1). However, approximately one-third of patients do not respond to this regimen (2), highlighting a need for different therapies against novel targets.

Tumor protein 53 (*TP53*) encodes the tumor suppressor p53, which participates in the regulation of the cell cycle, DNA repair, apoptosis, and senescence. Approximately 50% of human cancers have alterations in the *TP53* gene (3, 4). About 80% of *TP53* mutations are missense mutations and are located in the DNA-binding domain (DBD); 8 of these (R175, V157F, Y220C, G245, R248, R249, R273, and R282) account for ~28% of the total mutations in *TP53* (5). The *TP53* mutation is present in about 20% of patients with DLBCL (6) and is associated with a low treatment response rate (7, 8). Importantly, several hotspot mutations including those at residues 283, 248, 273, 175, 176, and 213—which are mainly located in the DBD, and specifically in the loop–sheet–helix and L3 motifs—are independent predictors of poor DLBCL outcome (9, 10). However, the mechanistic basis thereof is not fully understood. G/C-to-A/T mutations in *TP53* are considered high-risk hotspot mutations (11) and are similar to those induced by apolipoprotein B mRNA editing enzyme catalytic subunit 3s (APOBEC3s) family proteins (12).

Based on the above evidence, we speculated that APOBEC3 is responsible for *TP53* G/C-to-A/T mutations in DLBCL. To test this hypothesis, we analyzed the association between the *TP53* G/C-to-A/T mutation and APOBEC3B expression in clinical specimens from relapsed/refractory (R/R) DLBCL patients. Our results provide insight into the mechanistic basis for the *TP53* G/C-to-A/T mutation in DLBCL and reveal a new potential therapeutic target in the treatment of this disease.

## Materials and Methods

### Patients

A total of 61 patients (33 female and 28 male patients) diagnosed with *de novo* DLBCL by surgical biopsy of tumor tissue and treated with R-CHOP at the Second Affiliated Hospital of Zhejiang University School of Medicine and treated with R_CHOP from November 2016 to July 2018 were included in this study. The diagnosis of DLBCL was made independently by 2 experienced pathologists according to the 2016 World Health Organization classification; 19 cases were R/R DLBCL according to the criteria of Cheson et al. (13). This study was approved by the ethics committee of the hospital and was conducted in accordance with the principles of the Declaration of Helsinki. All patients provided written informed consent at enrollment.

### Detection of the *TP53* G/C-to-A/T Mutation in R/R DLBCL Clinical Specimens

Formalin-fixed, paraffin-embedded (FFPE) tumor tissue blocks were collected for each patient, and tumor tissue genomic DNA was extracted using the QIAamp DNA FFPE Tissue Kit (Qiagen) according to the manufacturer’s instructions. Peripheral blood samples were collected as matched normal samples. Total DNA of peripheral blood mononuclear cells (PBMCs) were extracted using the QIAamp DNA Blood Mini Kit (Qiagen) according to the manufacturer’s instructions. G/C-to-A/T mutations in *TP53* exon 8 were detected by differential DNA denaturation PCR (3D-PCR)-based Sanger sequencing as previously described (14), with some modifications (**Supplementary Material**).

### Cellular Localization of APOBEC3s

Immunofluorescence labeling of APOBEC3s-HA was performed as previously described (15). APOBEC3s-pEGFP detection is described in the **Supplementary Material**.

### DLBCL Database Analysis of APOBEC3A and APOBEC3B Expression

#### Oncomine DLBCL Database Analysis

As APOBEC3A and APOBEC3B were reported to potentially induce the *TP53* G/C-to-T/A mutation in several human cancers, we analyzed the expression status of APOBEC3A (12, 14) and APOBEC3B in DLBCL using the Oncomine database (http://www.oncomine.org) based on existing cancer microarray datasets. APOBEC3A and APOBEC3B expression data of 2 lymphoma groups, Brune lymphoma, and Compagno lymphoma, were extracted from GSE12453 and GSE12195 gene chips in the Gene Expression Omnibus (GEO) database (https://www.ncbi.nlm.nih.gov/geo/). Expression levels of APBOEC3A and APOBEC3B were compared between DLBCL and different types of normal cells as well as in DLBCL samples.

#### Gene Expression Profiling Interactive Analysis DLBCL Database Analysis

We searched the Gene Expression Profiling Interactive Analysis (GEPIA) database (http://gepia.cancer-pku.cn) to obtain expression data for *APOBEC3A* and *APOBEC3B* in DLBCL on box plots *via* Single Gene Analysis.

#### GEO DLBCL Database Analysis

DLBCL gene expression profiles and *APOBEC3A* and *APOBEC3B* expression data were obtained from the GEO database. Search results were sorted by the number of samples. Because the *APOBEC3A* and *APOBEC3B* expression data were derived from different sample types including frozen tumor tissue and FFPE tumor tissue, they were compared to frozen tissue and FFPE samples in GSE19246 to determine whether the sample source influenced the results.

### Detection of APOBEC3B in Refractory/Relapse DLBCL Samples

FFPE tumor tissue blocks were collected for each patient and APOBEC3B was detected by immunohistochemistry. Anti-APOBEC3B antibody was purchased from Abcam (Cambridge, MA, USA; cat. no. ab191695). The DAKO Envision+ System (Dako, Glostrup, Denmark; code K4002) was used for diaminobenzidine detection according to the manufacturer’s instructions.

### Construction of Plasmids and APOBEC3A/APOBEC3B-Inducible Cell Lines

#### Cell lines

Pfeiffer and OCI-LY10 DLBCL cell lines were purchased from the American Type Culture Collection (ATCC; Manassas, VA, USA) and cultured in RPMI1640 medium supplemented with 10% fetal bovine serum and penicillin/streptomycin. Human embryonic kidney (HEK)293 and 293T cell lines were purchased from ATCC and cultured in Dulbecco’s modified Eagle’s medium supplemented with 10% fetal bovine serum and penicillin/streptomycin. Cell lines were maintained in a humidified atmosphere of 5% CO_2_ at 37°C.

#### APOBEC3A/APOBEC3B-Inducible DLBCL Cell Lines

APOBEC3A/APOBEC3B-flag cDNA was cloned into the pLV-Ptight-puro vector (Clontech/Takara Bio, Dalian, China) to construct the inducible APOBEC3A/APOBEC3B-flag/pLV-Ptight-puro plasmid. Pfeiffer, OCI-LY10, 293T, and HEK293 cells were transfected using Polyjet *In Vitro* DNA Transfection Reagent (SignaGen, Frederick, MD, USA) according to the manufacturer’s protocol. The 293T cells were transfected with APOBEC3A/APOBEC3B-flag/pLV-ptight-puro vector along with PCL and 10A1. At 72 h after transfection, the supernatant containing viral particles was collected and purified by centrifugation and passed through a 0.45-μm filter. Then, the virus supernatant was stored at −80°C for the subsequent experiments. Pfeiffer, OCI-LY10, and HEK293 cells were seeded in a 6-well culture plate; after 24 h, the medium was replaced with fresh medium and culture supernatant with virus was added along with 8 μg/ml polybrene (Qiagen). After 12 h, the medium was replaced with fresh medium, and 48 h later, 3 μg/ml puromycin (Qiagen) was added to the medium to select infected clones. Inducible expression of APOBEC3A/APOBEC3B-flag was confirmed by Western blotting using an anti-flag antibody (Medical and Biological Laboratories, Nagoya, Japan; cat. no. M185-3L).

After puromycin selection for 14 days, the remaining cells were seeded at a concentration of 1 cell/well in a 96-well plate and cultured with a medium containing 4 μg/ml doxycycline (Sigma-Aldrich, St. Louis, MO, USA). Positive clones were used in the following steps.

### Detection of APOBEC3A/APOBEC3B-Induced G/C-to-A/T Mutations in *TP53* exon 8

After 14 days of induction with doxycycline, APOBEC3A/APOBEC3B-flag-inducible and pLV-pTight-puro cells were collected and total DNA was extracted using the QIAamp DNA mini kit (Qiagen). G/C-to-T/A mutations were detected and analyzed by 3D-PCR-based Sanger sequencing as described above.

### Analysis of Proliferation and Drug Sensitivity of p53-Mutant-derived DLBCL Cells

#### Colony Formation Assay

The colony formation assay was performed as previously described (16). Briefly, APOBEC3B-inducible DLBCL cells were induced with 4 μg/ml doxycycline for 14 days, after which the doxycycline was removed and the cells were cultured for another 24 h until APOBEC3B-Flag expression disappeared. APOBEC3B expression and control cells were seeded in 6-well culture plates containing semi-soft agarose at a concentration of 200 cells/well. The cells were cultured for another 14 days and colonies were subjected to Giemsa staining and counted under a microscope.

#### Construction of p53 Mutants (R273C and R282Q) and Doxorubicin Sensitivity Analysis in DLBCL

The PCW-CAS9 vector was modified to express wild-type and mutant p53. The mutant constructs (R273C and R282Q) were generated using the MutanBEST Kit (Takara, Dalian, China) according to the manufacturer’s instructions and transfected into Ly10 cells. Expression of wild-type and mutant p53 was induced with doxycycline and confirmed by Western blotting. Doxorubicin sensitivity was measured with the MTT assay (17).

### Statistical Analysis

Statistical analyses were performed with SPSS Statistics v20 (IBM, Armonk, NY, USA). Frequency tables and descriptive statistics (mean, median, minimum, and maximum) were used to summarize patient characteristics. The significance of differences between groups of patients was evaluated with the maximum likelihood chi-squared test and Fisher’s exact test for categorical variables and with the Mann–Whitney *U* test for continuous variables. Progression-free survival (PFS) was defined as time to disease progression, relapse, or death, and was estimated using the Kaplan–Meier method and compared using the log-rank test. The Kaplan–Meier method was used for univariate survival analysis. The multivariate Cox proportional hazard model was used to evaluate whether *TP53* mutation was an independent prognostic factor for PFS and overall survival. Colony formation data are reported as mean values ± SEM and were analyzed with the independent *t*-test. *APOBEC3A* and *APOBEC3B* gene expression data were compared and doxorubicin 50% inhibitor concentration (IC_50_) was analyzed using Prism 5 software (GraphPad, San Diego, CA, USA). *p*-values <0.05 were considered statistically significant.

## Results

### *TP53* exon 8 G/C-to-A/T Mutations Increased in R/R DLBCL Samples

**Figure 1:**
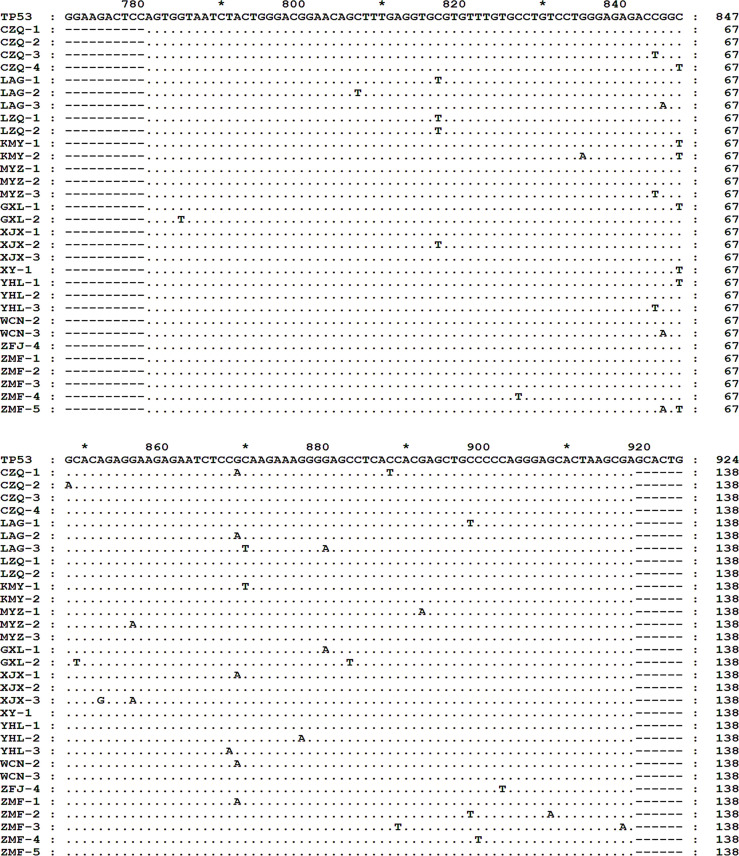
*TP53* exon 8 G/C-to-A/T mutations in R/R DLBCL. *TP53* exon 8 G/C-to-A/T mutation was detected in R/R DLBCL. Total DNA were extracted from FFPE DLBCL tissues and *TP53* exon 8 was amplified by 3D-PCR and sequenced by Sanger sequencing. Sequences were aligned and analyzed with the Clustal and Genedoc software. * indicates the number in the middle of the two numbers before and after.

**Figure 2:**
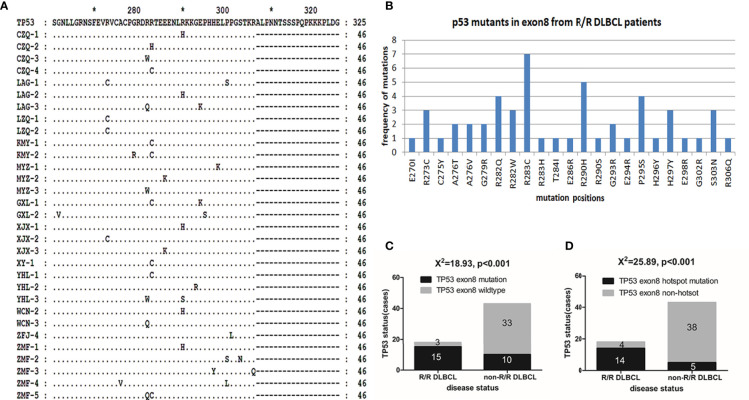
p53 mutants in R/R DLBCL. **(A)** p53 mutants were detected in R/R DLBCL. p53 protein sequences were translated with the Genedoc software based on DNA sequences and aligned with the Clustal and Genedoc software. **(B)** Distribution of p53 point mutants in R/R DLBCL. Numbers are the total number of patients in which a mutation was detected. **(C, D)** *TP53* mutation rates in R/R and non-R/R DLBCL. Frequencies were calculated as a percentage. Differences between groups of patients were assessed with the maximum likelihood chi-squared test.

Using the 3D-PCR-based direct sequencing method, G/C-to-A/T mutations were detected in clinical specimens of R/R DLBCL patients (**Figure 1** and **Supplementary Figure S1**). In most cases, the mutation resulted in changes in the amino acid sequence of the p53 protein (**Figure 2A** and **Supplementary Table S1**). Several mutations including R273C, R282W, R282Q, R283C, and R290H (**Figure 2B**) occurred more frequently than others; most of these (R273C, R282Q, R282W, and R283C) are previously reported hotspot mutations. *TP53* exon 8 G/C-to-A/T mutations were more frequent in R/R DLBCL samples (83.33%, 15/18) than in non-R/R DLBCL (23.26%, 10/43) samples (*χ* ^2^ = 18.93, *p* < 0.001) (**Figure 2C**). Hotspot mutations were detected in 14 of 18 R/R DLBCL patients, but only 5/43 non-R/R DLBCL patients (*χ* ^2^ = 25.89, *p* < 0.001) (**Figure 2D**).

**Figure 3:**
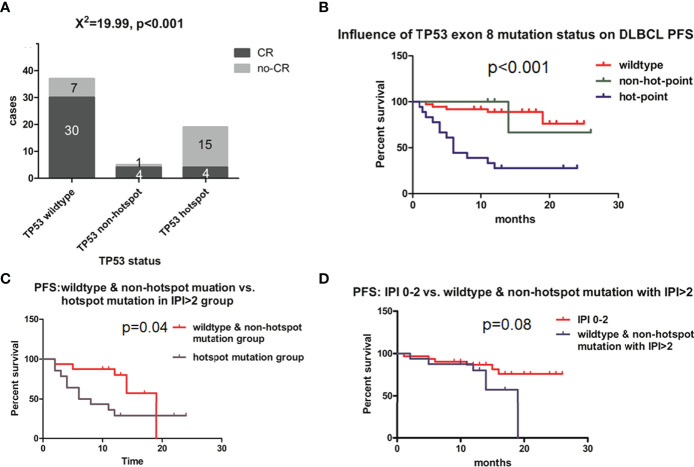
Effect of *TP53* exon 8 mutation on the clinical outcome of DLBCL patients. **(A)** CR rates in different groups based on *TP53* status. CR status was evaluated by positron emission tomography–computed tomography scanning according to Lugano response criteria for non-Hodgkin’s lymphoma. Differences between groups of patients were assessed with the maximum likelihood chi-squared test. **(B)** PFS of different *TP53* status groups. **(C)** PFS of IPI >2 group; patients with *TP53* hotspot mutations, wild-type *TP53*, and non-hotspot *TP53* mutations were compared. **(D)** Comparison of PFS between IPI 0–2 and IPI >2 groups with wild-type *TP53* and non-hotspot *TP53* mutations. Patients were followed up for 12 months until disease progression, relapse, or end of observation. PFS was defined as time to disease progression, relapse, or death, and was estimated with the Kaplan–Meier method and compared with the log-rank test.

The complete response (CR) rates of different groups based on *TP53* exon 8 status were calculated and compared at the 12-month follow-up. The overall CR rate for the study population was 68.90%, which was similar to a previously reported value (2). However, CR rates differed according to *TP53* mutation status. Among patients with wild-type *TP53* exon 8, the CR rate was 81.08% (30/37) (**Figure 3A**); by comparison, those with *TP53* exon 8 mutation had a CR rate of 28.57% (8/28) (*χ* ^2^ = 18.1, *p* < 0.01; data not shown). Patients harboring a hotspot mutation had the lowest CR rate (21.05% [4/19] vs. wild type, *χ* ^2^ = 18.97, *p* < 0.01; **Figure 3A**), whereas those with a non-hotspot mutation had a CR rate of 80% (4/5) (*p* = 0.028 vs. hotspot group [Fisher’s exact test] and *χ* ^2^ = 0, *p* = 1 vs. wild-type group; **Figure 3A**). Thus, the *TP53* wild-type and non-hotspot mutation groups combined had a higher CR rate than the *TP53* hotspot mutation group (*χ* ^2^ = 23.203, *p* < 0.01; data not shown). The hotspot mutation group had a median PFS of 6 months (95% confidence interval [CI]: 3.245–8.755; **Figure 3B**); meanwhile, PFS was not reached for the non-hotspot mutation and wild-type groups at the 12-month follow-up (**Figure 3B**). These 2 groups had similar demographic and clinical profiles except for sex ratio and B symptoms (**Table 1**).

**Table 1:** Clinical characteristics of patients in the *TP53* mutation groups.

Characteristic	*TP53* wild type and non-hotspot mutation group (*N* = 42)	*TP53* hotspot mutation group (*N* = 19)	*p*-value
Age, years			0.915
≤60	21 (50.0)	9 (47.4)	
61–70	12 (28.6)	7 (36.8)	
71–80	6 (14.3)	2 (10.5)	
>80	3 (7.1)	1 (5.3)	
Median (range)	60.5 (18–88)	61 (34–83)	
Sex			0.036
Male	15 (35.7)	13 (68.4)	
Female	27 (64.3)	6 (31.6)	
DLBCL subtype			0.803
GCB	14 (33.3)	5 (26.3)	
Non-GCB	28 (66.7)	14 (73.7)	
Stage			0.201
I	9 (21.4)	2 (10.5)	
II	9 (21.4)	1 (5.3)	
III	4 (9.5)	1 (5.3)	
IV	19 (45.2)	15 (78.9)	
B symptoms			0.029
Present	14 (33.3)	12 (63.2)	
Absent	28 (66.7)	7 (36.8)	
IPI			0.268
0	4 (9.5)	1 (5.3)	
1	11 (26.2)	2 (10.5)	
2	10 (23.8)	2 (10.5)	
3	7 (16.7)	7 (36.8)	
4	4 (9.5)	3 (15.8)	
5	5 (11.9)	4 (21.1)	

Values represent n (%) unless otherwise indicated.

Differences between groups were evaluated with the maximum likelihood chi-squared test and Fisher’s exact test for categorical variables and the Mann–Whitney U test for continuous variables.

DLBCL, diffuse large B-cell lymphoma; GCB, germinal center B-like; IPI, International Prognostic Index.

The *TP53* hotspot group had a greater proportion of patients with International Prognostic Index (IPI) >2 (high-intermediate and high risk) than the *TP53* wild-type and non-hotspot groups. We therefore examined the relationship between *TP53* mutation status and IPI and found that in the IPI >2 (high-intermediate and high risk) group, *TP53* mutation status also had a predictive value for PFS (**Figure 3C**, *p* = 0.04). Meanwhile, the PFS of *TP53* wild-type and non-hotspot mutation and the IPI > 2 group was similar to that of the IPI 0–2 group (*p* = 0.08; **Figure 3D**). These results indicate that *TP53* mutation status has a prognostic value that is not captured by the IPI for DLBCL.

To assess whether *TP53* mutation was an independent prognostic factor for shorter PFS, risk factors were first evaluated by univariate survival analysis with the Kaplan–Meier method (**Supplementary Table S2**). IPI, *TP53* mutation status, lactate dehydrogenase (LDH) activity, tumor stage, and sex were included in the multivariate Cox model. *TP53* hotspot mutation was the strongest independent predictor of PFS (hazard ratio [HR] = 5.146, 95% CI: 2.134–12.409; *p* < 0.0003), followed by *TP53* mutation (HR = 3.616, 95% CI: 1.49–8.773; *p* = 0.004). These data suggest that *TP53* mutation—especially in the hotspot—contributes to the poor outcome of DLBCL patients treated with R-CHOP.

### APOBEC3B but Not APOBEC3A Is Upregulated in DLBCL Compared to Normal Tissue

**Figure 4:**
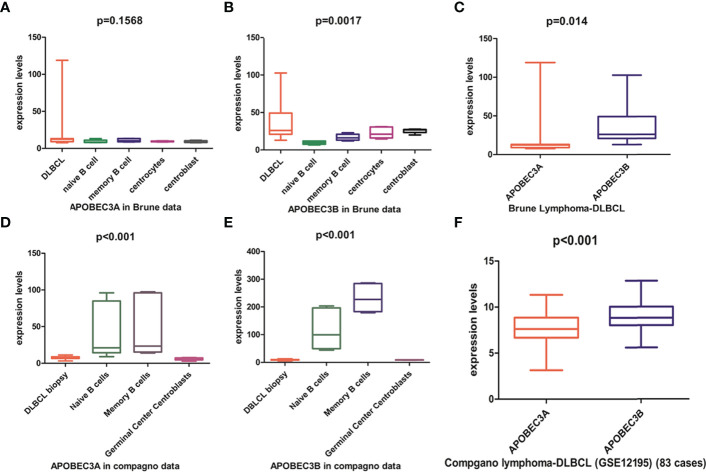
APOBEC3A and APOBEC3B expression in DLBCL based on the Oncomine online database. **(A, B)** APOBEC3A **(A)** and APOBEC3B **(B)** expression in the Brune lymphoma dataset including DLBCL, naïve B cells, memory B cells, centrocytes, and centroblasts. **(C)** Comparison of APOBEC3A and APOBEC3B expression in the Brune lymphoma dataset. **(D, E)** APOBEC3A **(D)** and APOBEC3B **(E)** expression in the Compagno lymphoma dataset including DLBCL, naïve B cell, memory B cell, and germinal center centroblasts. **(F)** Comparison of APOBEC3A and APOBEC3B expression in the Compagno lymphoma dataset.

APOBEC3A, APOBEC3B, APOBEC3F, and APOBEC3G of the APOBEC3s family exhibit cytidine deaminase activity that induces G/C-to-A/T mutation in a single DNA strand. APOBEC3F and APOBEC3G cause hypermutation in the human immunodeficiency virus (HIV) genome but are localized in the cytoplasm (**Supplementary Figure S2**). APOBEC3A is localized in both the cytoplasm and the nucleus, whereas APOBEC3B is predominantly localized in the nucleus (**Supplementary Figure S2**) with access to genomic DNA. Previous studies have shown that APOBEC3A and APOBEC3B may contribute to host DNA mutation. Analysis of the Brune lymphoma dataset in the Oncomine database revealed that the *APOBEC3A* mRNA level in DLBCL was similar to (**Figure 4A**) whereas the *APOBEC3B* mRNA level was higher than (**Figure 4B**) that in normal tissue. Similar trends in *APOBEC3B* and *APOBEC3A* expression in DLBCL were observed in The Cancer Genome Atlas (TCGA)-based GEPIA database (data not shown), but not in the Compagno lymphoma DLBCL dataset of TCGA (**Figures 4D, E**). We also found that APOBEC3B was more highly expressed than APOBEC3A in DLBCL tissues in both the Brune and Compagno lymphoma datasets (**Figures 4C, F**).

*APOBEC3A* and *APOBEC3B* expression levels in DLBCL were further compared in the GEO database. The results were sorted by the number of samples from highest to lowest, and the top 13 results were analyzed. The *APOBEC3A* expression level was higher than that of *APOBEC3B* in 3/13 datasets including the largest one (**Supplementary Figures S3A, D, I**), whereas in 10/13 datasets, the *APOBEC3B* expression level was higher (**Supplementary Figure S3**). In the dataset with the largest number of samples (GSE117556), RNA was extracted from FFPE tumor tissue; therefore, *APOBEC3A* and *APOBEC3B* expression levels were analyzed in different samples in GSE19246, which contains both frozen and FFPE tumor tissue. *APOBEC3A* expression level was similar between the 2 sample sources (**Supplementary Figure S4A**), but the *APOBEC3B* expression level was higher in frozen tumor tissue than in the FFPE sample (**Supplementary Figure S4B**). These results indicate that analyzing the *APOBEC3B* expression level in FFPE tumor tissue sample may result in an underestimate. Additionally, the *APOBEC3B* expression level was higher than that of *APOBEC3A* in most frozen tumor tissue samples. In summary, the analysis of online databases showed that *APOBEC3B* was more highly expressed than *APOBEC3A* in DLBCL.

### APOBEC3B Level Is Higher in R/R DLBCL Than in Non-R/R DLBCL

**Figure 5:**
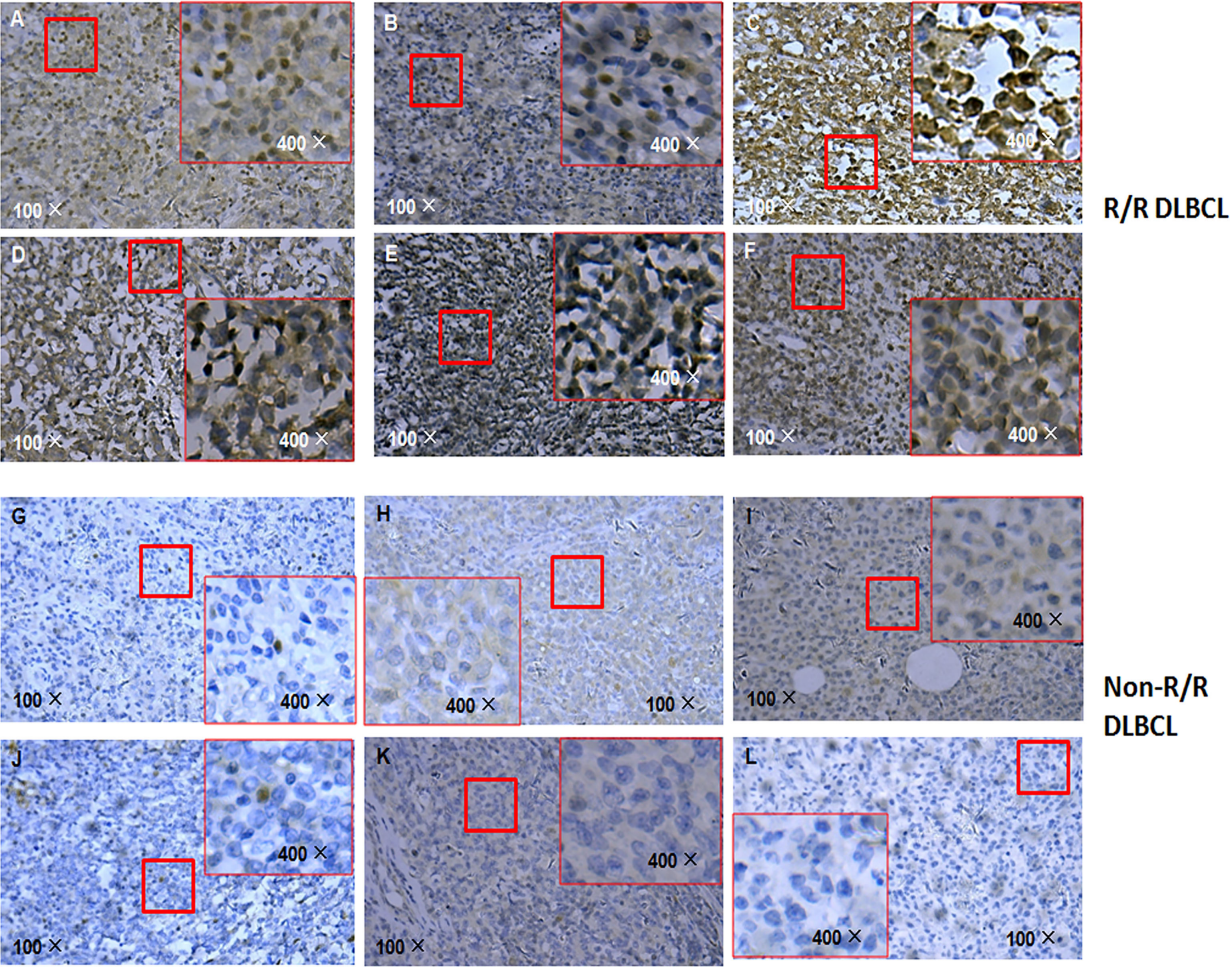
APOBEC3B expression in R/R DLBCL and non-R/R DLBCL samples detected by immunohistochemistry. **(A–F)** R/R samples. **(G–L)** Non-R/R samples.

Given that APOBEC3B induces G/C-to-A/T mutation in human cancers including breast cancer and is upregulated in DLBCL, we compared the level of APOBEC3B protein in R/R and non-R/R DLBCL samples by immunohistochemistry. Interestingly, APOBEC3B protein level was higher in R/R DLBCL (**Figures 5A–F**) than in non-R/R DLBCL (**Figures 5G–L**) samples. As expected, APOBEC3B protein was predominantly localized in the nucleus. Based on the analysis of the APOBEC3B expression level stratified by *TP53* mutation status using a cutoff value for an APOBEC3B positive rate of 20%, we found that the APOBEC3B protein level was higher in the *TP53* mutation group (58.33%) than in the *TP53* wild-type group (27.03%) (*χ* ^2^ = 4.657 *p* = 0.038) (**Supplementary Table S3**). Moreover, the APOBEC3B positive rate was higher in the *TP53* hotspot mutation group (68.42%) than in the *TP53* wild-type and non-hotspot groups (26.19%) (*χ* ^2^ = 9.776, *p* = 0.004) (**Supplementary Table S4**). These data suggest that APOBEC3B overexpression is associated with *TP53* mutation—especially those in the hotspot—in R/R DLBCL.

### APOBEC3B Induces G/C-to-A/T Mutation in DLBCL Cell Lines

**Figure 6:**
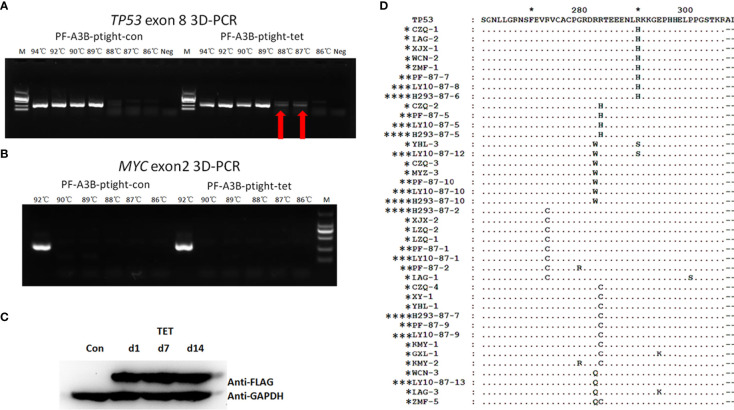
APOBEC3B induces *TP53* exon 8 mutations *in vitro*. **(A)** 3D-PCR amplification of *TP53* exon 8 in APOBEC3B-inducible Pfeiffer cells (right) and control cells (left). Red arrows indicate DNA fragments that amplified at a denaturing temperature of 88°C and 87°C. PCR products were separated by agarose gel electrophoresis (top). APOBEC3B-flag expression was confirmed by Western blotting using an anti-flag antibody (bottom). Con, cell lysis before induction with doxycycline; d1, cell lysis after induction with doxycycline for 1 day; d7, cell lysis after induction with doxycycline for 7 days; d14, cell lysis after induction with doxycycline for 14 days; M, DNA ladder marker; Neg, negative control; PF, Pfeiffer. Denaturation temperatures in 3D-PCR ranged from 92°C to 87°C. **(B)** 3D-PCR amplification of MYC exon 2 in APOBEC3B-inducible Pfeiffer cells. PCR products were separated by agarose gel electrophoresis (top). Expression of APOBEC3B-flag was confirmed by Western blotting using an anti-flag antibody (bottom). **(C)** Detection of APOBEC3B-flag by Western blotting in APOBEC3B-inducible cells. **(D)** Comparison of *in vitro* APOBEC3B-induced p53 mutants with R/R DLBCL samples. P53 sequences were analyzed using Clustal and Genedoc software. *DLBCL patients. **APOBEC3B-inducible Pfeiffer cells. ***APOBEC3B-inducible OCI-LY10 cells. **** APOBEC3B-inducible HEK293 cells.

To further investigate whether overexpressed APOBEC3B can induce *TP53* mutation in DLBCL, we performed an *in vitro* assay using DLBCL cell clones with inducible APOBEC3B expression. APOBEC3B-flag expression was confirmed by Western blotting following induction with doxycycline (**Figure 6C**). *TP53* exon 8 was amplified by 3D-PCR and c-MYC exon 2, which is less frequently mutated in DLBCL (18, 19), and was also amplified as a control. As the denaturing temperature was decreased from 94°C to 87°C, the *TP53* exon 8 fragment was detected at 88°C and 87°C in APOBEC3B-inducible cells but not in control cells (**Figure 6A**). Meanwhile, the *MYC* exon 2 fragment was not detected at denaturing temperatures <94°C (**Figure 6B**). Additionally, in DLBCL cells overexpressing APOBEC3A, the *TP53* exon 8 fragment was not detected by 3D-PCR at a denaturing temperature of 89°C (**Supplementary Figure S5**). Single-clone sequencing of *TP53* exon 8 PCR products at 87°C revealed that >20% of clones harbored >1 G/C-to-A/T mutation compared to the control and the 94°C PCR products. The major mutation patterns were TG and GC; the mutation pattern and sites were similar to that of DLBC samples (**Figure 6D**). Further analysis showed that there were several known hotspot amino acid mutations including R273C, R282Q, R282W, and R283W (**Figure 6D**). These data suggest that APOBEC3B can induce the *TP53* G/C-to-A/T mutation—including in the hotspot—in DLBCL cells.

### APOBEC3B-Induced p53 Mutation Promotes Cell Proliferation and Doxorubicin Resistance

**Figure 7:**
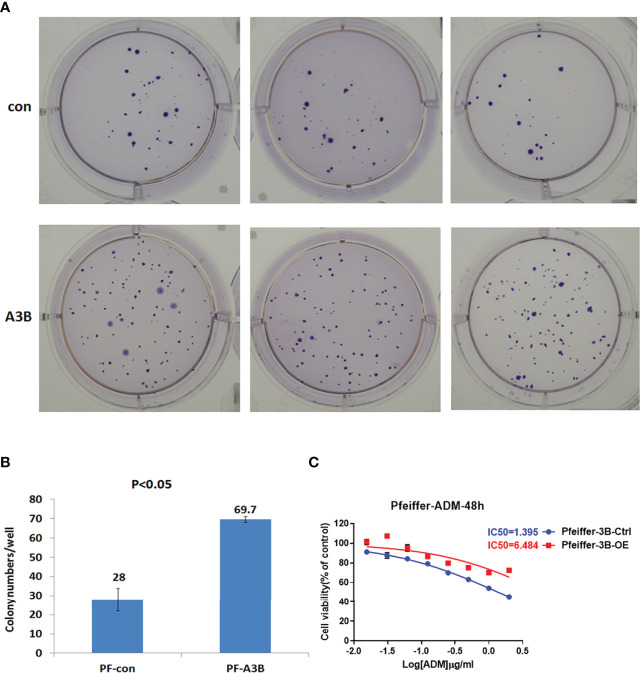
APOBEC3B-induced proliferation and drug resistance in DLBCL cells. **(A, B)** Colony formation after induction of APOBEC3B expression. A3B inducible, APOBEC3B-Flag/pLV-Ptight-puro vector-transfected Pfeiffer cells; Con, pLV-Ptight-puro vector-transfected Pfeiffer cells; PF-A3B, APOBEC3B-Flag/pLV-Ptight-puro vector-transfected Pfeiffer cells; PF-con, pLV-Ptight-puro vector-transfected Pfeiffer cells. **(C)** Doxorubicin resistance after induction of APOBEC3B expression. ADM, doxorubicin; Pfeiffer-3B-Ctrl, pLV-Ptight-puro vector-transfected Pfeiffer cells; Pfeiffer-3B-OE, APOBEC3B-Flag/pLV-Ptight-puro vector-transfected Pfeiffer cells.

**Figure 8:**
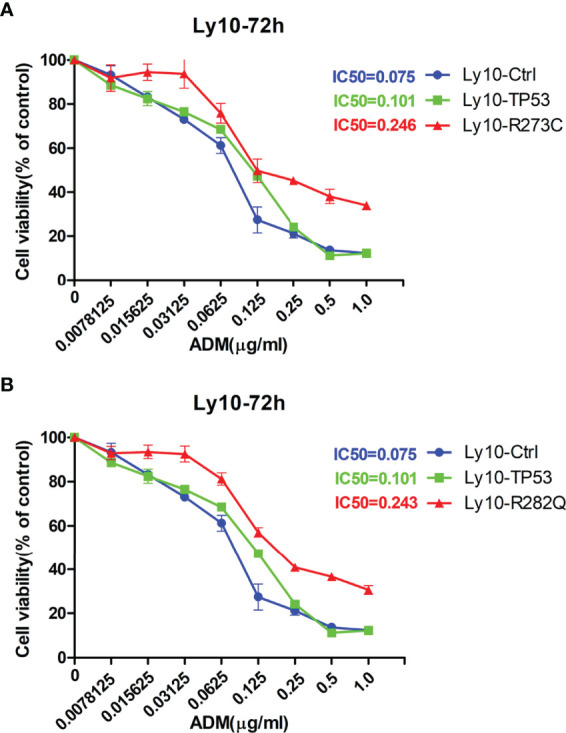
p53 mutants (R273C and R282Q) induce doxorubicin resistance. **(A, B)** Effect of R273C **(A)** and R282Q **(B)** mutation on doxorubicin sensitivity in Ly10 cells. ADM, doxorubicin; Ly10-Ctrl, empty vector-transfected Ly10 cell; Ly10-R273C, p53 R273C mutant-transfected Ly10 cells; Ly10-R282Q, p53 R282Q mutant-transfected Ly10 cells; Ly10-TP53, wild-type p53-transfected Ly10 cells.

Given our finding that APOBEC3B induced *TP53* mutation, we examined the effect of this mutation on proliferation and drug sensitivity of APOBEC3B-expressing Pfeiffer DLBCL cells. The results of the semi-soft agar colony formation assay showed that after the induction of APOBEC3B expression for 14 days, cells generated more colonies than the control group despite APOBEC3B expression being restored (**Figures 7A, B**). APOBEC3B-expressing cells showed resistance to doxorubicin, with a 4.65-fold increase in IC_50_ compared to the control (6.484 vs. 1.395 μg/ml) (**Figure 7C**). The results of the *in vitro* doxorubicin sensitivity assay using Ly10 cells (wild-type *TP53* DLBCL cell line) expressing mutant p53 showed that the R273C and R282Q mutants had reduced sensitivity to doxorubicin, with 2.28- and 2.23-fold increases in IC_50_, respectively (**Figure 8**). These data suggest that APOBEC3B-induced mutant p53 promotes proliferation and confers drug resistance in DLBCL cells.

## Discussion

The results of this study demonstrate that APOBEC3B induces p53 mutation and may confer drug resistance in DLBCL. The rate of *TP53* mutation in DLBCL is 20%–30%, with similar frequencies of the germinal center B-cell and activated B-cell subtypes (7–9). In the present study, 3D-PCR-based sequencing identified *TP53* exon 8 mutations in 35.9% of the cohort, which is higher than in previous reports. Even in non-R/R DLBCL cases, the TP53 exon 8 mutation rate was 23.26%. This may be explained by selective amplification of DNA fragments containing the G/C-to-A/T mutation (20). Thus, 3D-PCR detected the G/C-to-A/T mutation with higher sensitivity than standard PCR. A shortcoming of this method is that it misses other mutation types; however, as G/C to A/T is the main *TP53* mutation and accounts for most hotspot mutations (10), 3D-PCR is a valuable method for *TP53* mutation detection in DLBCL.

Given its function as a tumor suppressor, mutations in *TP53* predict poor prognosis in acute myelogenous leukemia and chronic lymphocytic leukemia in National Comprehensive Cancer Network guidelines and in multiple myeloma in the Mayo Clinic Stratification for Myeloma and Risk-Adapted Therapy system. It has been reported that *TP53* mutation is also a prognostic factor for a poor outcome in DLBCL (7–9). However, other studies failed to demonstrate a correlation between *TP53* mutation and prognosis (21, 22). In the present study, we found that *TP53* exon 8 G/C-to-A/T mutation frequency was higher in R/R DLBCL patients than in non-R/R DLBCL patients. Most were missense mutations that caused an amino acid change. Patients with *TP53* mutation had a lower CR rate than those with wild-type *TP53* when treated with the R-CHOP regimen. *TP53* hotspot mutations were able to distinguish patients with IPI > 2, who had a worse outcome than patients with high-risk IPI but wild-type and non-hotspot *TP53*, who had an outcome similar to the IPI 0–2 group. The multivariate Cox model showed that *TP53* hotpot mutation was a stronger prognostic factor for PFS than IPI, LDH activity, tumor stage, and sex. Consistent with previous reports, our data showed that *TP53* mutation was an independent prognostic factor for poor outcome in DLBCL. However, as we only sequenced and analyzed exon 8, the *TP53* mutation rate may have been underestimated; *TP53* mutations in other exons need to be examined to confirm this association.

About one-third of *TP53* mutations were clustered in 5 hotspot residues—namely, p.R175, p.G245, p.R248, p.R273, and p.R282 (23). Most previous studies did not evaluate the differential prognostic value of *TP53* mutation at different positions in DLBCL. Mutations in the DBD—especially in the loop–sheet–helix motif and L3 regions—were shown to be associated with worse survival (8). Codons 248, 273, 175, 176, and 213 of the p53 protein have the highest mutation frequency, which is associated with poor prognosis (9). Thus, hotspot mutations may be more important for p53 activity and have greater prognostic value than non-hotspot mutations. We found that some hotspot mutations including those in codons 273, 282, and 283 were higher in R/R DLBCL than in non-R/R DLBCL. Patients with non-hotspot mutations had a CR rate and PFS similar to patients with wild-type *TP53*; however, this requires confirmation in a larger cohort. Hotspot mutations but not non-hotspot mutations were strongly associated with lower CR rate and shorter PFS in DLBCL patients, and were previously shown to affect the DNA-binding activity of the p53 protein, thereby impairing the regulation of target genes (24, 25). Some of these hotspot p53 mutants were also responsible for gain of function in carcinogenesis and drug resistance (26, 27). For example, the R273C and R273H mutants conferred cancer cells with a more aggressive phenotype and enhanced resistance to DNA-damaging drugs (28). The *TP53* missense mutations R273H and R282W were also reported to exert a dominant-negative effect and caused drug resistance in AML cells (29). We found that hotspot mutations rather than all *TP53* mutation sites were associated with worse survival in DLBCL patients. Which of these mutations have a greater prognostic value and how they influence the role of p53 in tumorigenesis and drug resistance remain to be determined.

*TP53* mutation may confer resistance to chemotherapy agents such as doxorubicin (29) and cisplatin (30, 31), which are typically used to treat DLBCL. However, the underlying mechanism is not well understood, which has prevented the development of therapeutic strategies to overcome mutant p53-mediated drug resistance. G/C to A/T is the most common *TP53* mutation type, especially at mutation hotspots. Most known carcinogens such as polycyclic aromatic hydrocarbons (benzo[a]pyrene), aristolochic acid, aflatoxin B1, vinyl chloride, and 3-nitrobenzanthrone do not induce G/C-to-A/T mutation (32), although ultraviolet radiation was reported to induce CC-to-TT mutation in skin cancer (33). However, the mechanism through which most *TP53* G/C-to-A/T mutations arise is unclear. Our group and others previously demonstrated that some APOBEC3 family members could induce the G/C-to-A/T mutation in the genome of viruses including HIV (34, 35), HTLV-1 (36), and HBV (15, 37, 38). APOBEC3B is among the most widely studied factors related to G/C-to-A/T mutations in various human cancers (14, 39, 40). APOBEC3s were shown to be upregulated by interferon (41), which is an important cytokine involved in inflammation; chronic inflammation may contribute to lymphoma carcinogenesis. Based on our findings, we propose that APOBEC3s—especially APOBEC3B—are responsible for the *TP53* G/C-to-A/T mutation.

Among the APOBEC3 family members, APOBEC3B is mainly localized in the nucleus; APOBEC3G, APOBEC3F, and APOBEC3DE are present in the cytoplasm; and APOBEC3A and APOBEC3C are localized in both compartments (15, 37). A bioinformatic analysis of APOBEC3 expression in DLBCL revealed that APOBEC3B but not APOBEC3A was upregulated in DLBCL in several lymphoma databases, suggesting that it is responsible for *TP53* mutations in DLBCL.

Using an inducible expression system, we overexpressed APOBEC3B in DLBCL cells and found that the G/C-to-A/T mutation was induced in *TP53* exon 8. These *in vitro* APOBEC3B-induced mutation patterns including hotspot mutants were the same as those observed in clinical R/R DLBCL samples. Overexpression of APOBEC3A did not induce the G/C-to-A/T mutation in *TP53*. Thus, APOBEC3B and not APOBEC3A may be responsible for the *TP53* G/C-to-A/T mutation in DLBCL, although the molecular details remain to be elucidated. The APOBEC3B-induced mutants enhanced the proliferation of DLBCL cells and conferred resistance to the CHOP component doxorubicin, which has been previously reported (29). Thus, APOBEC3B-induced p53 mutants may be responsible for R/R DLBCL. As APOBEC3B targets different DNA sequences in different cells, additional research is needed to determine the sequence and associated factors in R/R DLBCL. Recently, the p53 protein was shown to regulate APOBEC3B3 expression, which increased APOBEC signature mutations in p53-defective cells (42). Thus, the crosstalk between p53 and APOBEC3B in carcinogenesis and drug resistance warrants further study.

As APOBEC3B has been shown to be associated with mutations in genomic DNA, tumorigenesis, and drug resistance, it is a potential therapeutic target for cancer treatment. Inhibiting APOBEC3B *via* modified single-stranded DNA and thereby reducing the risk of genomic mutations (43–45) and targeting uracil DNA glycosylases to selectively inhibit tumor cells with high APOBEC3B expression (46) have been described as possible strategies. However, it is unclear whether the former can reduce the frequency of *TP53* mutations in DLBCL and the latter can prevent R/R in DLBCL.

## Conclusion

In this study, we demonstrated that aberrantly activated APOBEC3B can induce *TP53* G/C-to-A/T mutations in DLBCL, which may lead to proliferation and drug resistance and may contribute to R/R DLBCL. As a DNA mutator, APOBEC3B is a potential therapeutic target to reduce the rate of *TP53* mutation and improve the prognosis of DLBCL patients. *TP53* mutants—especially those in hotspots—have a prognostic value for DLBCL treated with R-CHOP. We also provided a more sensitive method for detecting *TP53* mutation in tumor tissue DNA, which may be helpful for further study of *TP53* mutations in malignancies beyond DLBCL. In conclusion, our findings provide insight into the mechanism underlying *TP53* mutation in DLBCL as well as a potential target for overcoming drug resistance in this disease.

## Data Availability Statement

The datasets presented in this study can be found in online repositories. The names of the repository/repositories and accession number(s) can be found in the article/**Supplementary Material**.

## Ethics Statement

The studies involving human participants were reviewed and approved by the Ethics Committee of the Second Affiliated Hospital of Zhejiang University School of Medicine. The patients/participants provided their written informed consent to participate in this study.

## Author Contributions

XZZ and RX designed the experiments. XZZ, ZW, TY, XiaL, and YL performed the experiments. JL and XiuL contributed to the analysis of pathological data. YX, LH, XH, WW, GX, and XHZ collected the clinical data. QL and YF contributed to the collection of patient material. XZZ and ZW contributed to the analysis of the data. YH contributed to the data analysis and revision. XZZ, RX, and WQ contributed to the writing of the manuscript. All authors contributed to the article and approved the submitted version.

## Funding

This work was supported by a grant from the National Natural Science Foundation of China (81000895 to XZZ), a grant from the Zhejiang Provincial Natural Science Foundation of China (LY14H160032 to XZZ), and a grant from the Leukemia Research Innovative Team of Zhejiang Province (2011R50015 to XZZ). This work was also partly supported by a grant from the Zhejiang Provincial Natural Science Foundation of China (LQ22H080007 to ZW).

## Conflict of Interest

The authors declare that the research was conducted in the absence of any commercial or financial relationships that could be construed as a potential conflict of interest.

## Publisher’s Note

All claims expressed in this article are solely those of the authors and do not necessarily represent those of their affiliated organizations, or those of the publisher, the editors and the reviewers. Any product that may be evaluated in this article, or claim that may be made by its manufacturer, is not guaranteed or endorsed by the publisher.
