# Supplementary material for: Aberrantly Activated APOBEC3B Is Associated With Mutant p53-Driven Refractory/Relapsed Diffuse Large B-Cell Lymphoma
Source: Front Immunol. 2022 May 3;13:888250. doi: 10.3389/fimmu.2022.888250 (PMC9112561; doi:10.3389/fimmu.2022.888250)
Supplement: Supplementary file 1 [file DataSheet_1.zip › supplementary/Supplementary method.docx]

**Supplementary method:**

Constructions of plasmids and APOBEC3A-inducible cell lines: APOBEC3A-flag cDNA was cloned into pLV-Ptight-puro vector (Clontech, Takara Bio, China) to construct inducible APOBEC3A-flag/pLV-Ptight-puro plasmid. Pfeiffer was transfected using Polyjet In Vitro DNA Transfection Reagent (SignaGen, USA) according to manufacturer’s protocol. The 293T cells were transfected with APOBEC3A-flag/pLV-ptight-puro vector as well as PCL and 10A1. Seventy-two hours after transfection, virus supernatants were collected and purified by centrifuge and 0.45 um filter. Then the virus supernatant was stored at -80℃ for the subsequent experiments. Pfeiffer cell was seeded in 6-well culture plate, 24 hours later the medium was replaced with fresh medium and virus supernatant were added into the medium as well as 8ug/ml polybrene (Qiagen, USA). After infection for 12 hours, the medium was replaced with fresh medium. Forty-eight hours later, 3ug/ml puromycin (Qiagen, USA) was added to the medium for selection of infected clones. Inducible expression of APOBEC3A-flag was confirmed by Western blot using anti-flag antibody (Cat No. M185-3L, MBL, Japan).

After selection by puromycin for 14 days, the remainder cells were seeded at concentration of one cell/well in 96-well plate and checked by Western Blot after cultured with medium containing 4ug/ml doxycycline (Sigma, USA). Positive clones were maintained and used in the following steps.

Detection of APOBEC3A-induced TP53 exon8 G/C to A/T mutations: After 14days of induction, the APOBEC3A-flag-inducible cells as well as empty pLV-pTight-puro vector cells were collected and total DNA were extracted using Qiagen DNA mini kit. The G/C to T/A mutations were detected and analyzed by 3D-PCR based Sanger sequencing method as described previously.

Figure legends:

Figure S1: Sequencing results of TP53 exon8 hotspot mutations in R/R DLBCL.

S1A: C817T/R273C mutation sequencing result.

S1B: C844T/R282W mutation sequencing result.

S1C: G845A/R282Q mutation sequencing result.

S1D: C847T/R283C mutation sequencing result.

S1E: G869A/R290H mutation sequencing result.

Wild type: Wild type sequence from PBMC DNA. Mutation type: Mutation type sequence from DLBCL samples DNA.

The TP53 exon8 was amplified by 3D-PCR from DLBCL FFPE DNA and by regular PCR from PBMC DNA, and then were sequenced by Sanger sequencing method. Wild type sequences and mutation sequences were analyzed by chromas software.

Figure S2: Intracellular localization of APOBEC3s.

S2A: Intracellular localization of APOBEC3s in Hek293 cell (immunofluorescence). APOBEC3s-HA were transiently expressed in Hek293 cells, then the cells were fixed and APOBEC3s were detected using anti-HA antibody and rhodamine-conjugated anti-mouse antibody.

S2B: Intracellular localization of APOBEC3s in Hek293 cell (pEGFP fusion protein).

Figure S3: Compare of APOBEC3A and APOBEC3B expression in DLBCL based on GEO database.

GSE: different data numbers; cases: number of cases included.

Gene expression profiling data of DLBCL were obtained from the GEO online database. Expression data of APOBEC3A and APOBEC3B were obtained from these data, and then were compared by GraphPad Prism5 software.

Figure S4: Compare of APOBEC3A and APOBEC3B between frozen tumor tissue and formalin-fixed, paraffin-embedded (FFPE) tumor tissue in GSE19246.

Expression data of APOBEC3A and APOBEC3B were obtained from GSE19246, and then were compared by GraphPad Prism5 software.

Figure S5: 3D-PCR amplified of TP53 exon8 from APOBEC3A-inducible Pfeiffer cells.

S5A: 3D-PCR amplification of TP53 exon8 in APOBEC3A-inducible Pfeiffer cell (Right) and control cell (Left). PCR products were separated in agarose gel electrophoresis. M: DNA ladder marker. PF: Pfeiffer. Neg: negative control. 92℃ to 87℃ were different denature temperatures in 3D-PCR.

S5B: Expression of APOBEC3A-flag was confirmed by Western Blot via anti-flag antibody.
