# Supplementary material for: Aberrantly Activated APOBEC3B Is Associated With Mutant p53-Driven Refractory/Relapsed Diffuse Large B-Cell Lymphoma
Source: Front Immunol. 2022 May 3;13:888250. doi: 10.3389/fimmu.2022.888250 (PMC9112561; doi:10.3389/fimmu.2022.888250)
Supplement: Supplementary file 1 [file DataSheet_1.zip › supplementary/Table S1.docx]

| Patients' ID | DNA sequence change | Amino acid change |
| --- | --- | --- |
| CZQ | G869A | R290H |
|  | G830A | C277Y |
|  | G848A | R283H |
|  | G826A | A276T |
|  | C844T | R282W |
|  | C847T | R283C |
| LAG | C827T+C898T | R273C+P300S |
|  | G869A | R290H |
|  | G845A+G880A | R282Q+E294R |
|  | C851T | T184I |
|  | G877A+C889T+G908A | G293R+H297Y+S303N |
| LZQ | C817T | R273C |
| KMY | G826A | A276T |
|  | G824A | C275Y |
|  | C884T | P295S |
|  | C847T | R283C |
|  | G835A+C847T | G27R+R283C |
| MYZ | G892A | E298R |
|  | C898T | P300S |
|  | G845A | E286R |
|  | C844T | R282W |
| GXL | C847T+G880A | R283C+E294R |
|  | G880A+G908A | E294R+S303N |
|  | C884T | P295S |
|  | C286T | G262V |
| XJX | G869A | R290H |
|  | C847T | R283C |
|  | G806A | F270I |
|  | G845A | E286R |
|  | C827T | A276V |
| XY | C847T | R283C |
| YHL | C847T | R283C |
|  | G877A | G293R |
|  | G820A | V274I |
|  | C844T+C868A | R282W+R290S |
| WCN | G845A | R282Q |
|  | G869A | R290H |
| ZFJ | C902T | P301L |
| ZMF | G869A | R290H |
|  | C898T+G908A | P300S+S303N |
|  | C889T+G917A | H297U+R306Q |
|  | C827T+C899T | A276V+P300L |
|  | G845A+C847T | R282Q+R283C |

**Table S1: *TP53* exon8 mutations in R/R DLBCL samples.**

Genomic DNA was extracted from R/R DLBCL FFPE samples. *TP53* exon8 G/C to A/T mutations was detected through 3D-PCR based Sanger sequencing. The sequences were aligned and analyzed with Clustal and Genedoc software.
