# Supplementary material for: Aberrantly Activated APOBEC3B Is Associated With Mutant p53-Driven Refractory/Relapsed Diffuse Large B-Cell Lymphoma
Source: Front Immunol. 2022 May 3;13:888250. doi: 10.3389/fimmu.2022.888250 (PMC9112561; doi:10.3389/fimmu.2022.888250)
Supplement: Supplementary file 1 [file DataSheet_1.zip › supplementary/Table S2.docx]

| Variables | Hazard ratio | 95% CI | P |
| --- | --- | --- | --- |
| Stage III/VI | 3.791 | 1.114-12.901 | 0.033 |
| Male | 3.729 | 1.444-9.628 | 0.007 |
| High LDH level | 3.192 | 1.214-8.392 | 0.019 |
| IPI >2 | 3.366 | 1.291-8.78 | 0.013 |
| TP53 mutation | 3.616 | 1.49-8.773 | 0.004 |
| TP53hotspot mutation | 5,146 | 2.134-12.409 | 0.0003 |

Table S2. Univariate analysis of *TP53* mutation and clinical features in diffuse large B-cell lymphoma
