# Supplementary material for: Aberrantly Activated APOBEC3B Is Associated With Mutant p53-Driven Refractory/Relapsed Diffuse Large B-Cell Lymphoma
Source: Front Immunol. 2022 May 3;13:888250. doi: 10.3389/fimmu.2022.888250 (PMC9112561; doi:10.3389/fimmu.2022.888250)
Supplement: Supplementary file 1 [file DataSheet_1.zip › supplementary/table S3.docx]

|  | *TP53* mutation | *TP53* wild type |  |
| --- | --- | --- | --- |
| APOBEC3B>20% | 14(58.33%) | 10(27.03%) | 24 |
| APOBEC3B<20% | 10(41.67%) | 27(72.97%) | 37 |
| total | 24 | 37 | 61 |

X2=4.657 p=0.038

Table S3. Distribution of APOBEC3B protein in *TP53* mutation and wild type groups (IHC).

Frequencies were calculated as percentage. Differences between compared groups of patients were assessed by Maximum Likelihood Chi-square test using IBM SPSS Statistics 20.
