# Supplementary material for: Aberrantly Activated APOBEC3B Is Associated With Mutant p53-Driven Refractory/Relapsed Diffuse Large B-Cell Lymphoma
Source: Front Immunol. 2022 May 3;13:888250. doi: 10.3389/fimmu.2022.888250 (PMC9112561; doi:10.3389/fimmu.2022.888250)
Supplement: Supplementary file 1 [file DataSheet_1.zip › supplementary/table s4.docx]

|  | *TP53* hotspot mutation | *TP53* wildtype & non-hotspot mutation |  |
| --- | --- | --- | --- |
| APOBEC3B>20% | 13(68.42%) | 11(26.19%) | 24 |
| APOBEC3B<20% | 6(31.58%) | 31(73.81%) | 37 |
| total | 19 | 42 | 61 |

X2=9.776 p=0.004

Table S4. Distribution of APOBEC3B protein in TP53 hotspot mutation group and wildtype & non-hotspot mutation group (IHC).

Frequencies were calculated as percentage. Differences between compared groups of patients were assessed by Maximum Likelihood Chi-square test using IBM SPSS Statistics 20.
