# Supplementary figures and images for: Aberrantly Activated APOBEC3B Is Associated With Mutant p53-Driven Refractory/Relapsed Diffuse Large B-Cell Lymphoma
Source: Front Immunol. 2022 May 3;13:888250. doi: 10.3389/fimmu.2022.888250 (PMC9112561; doi:10.3389/fimmu.2022.888250)

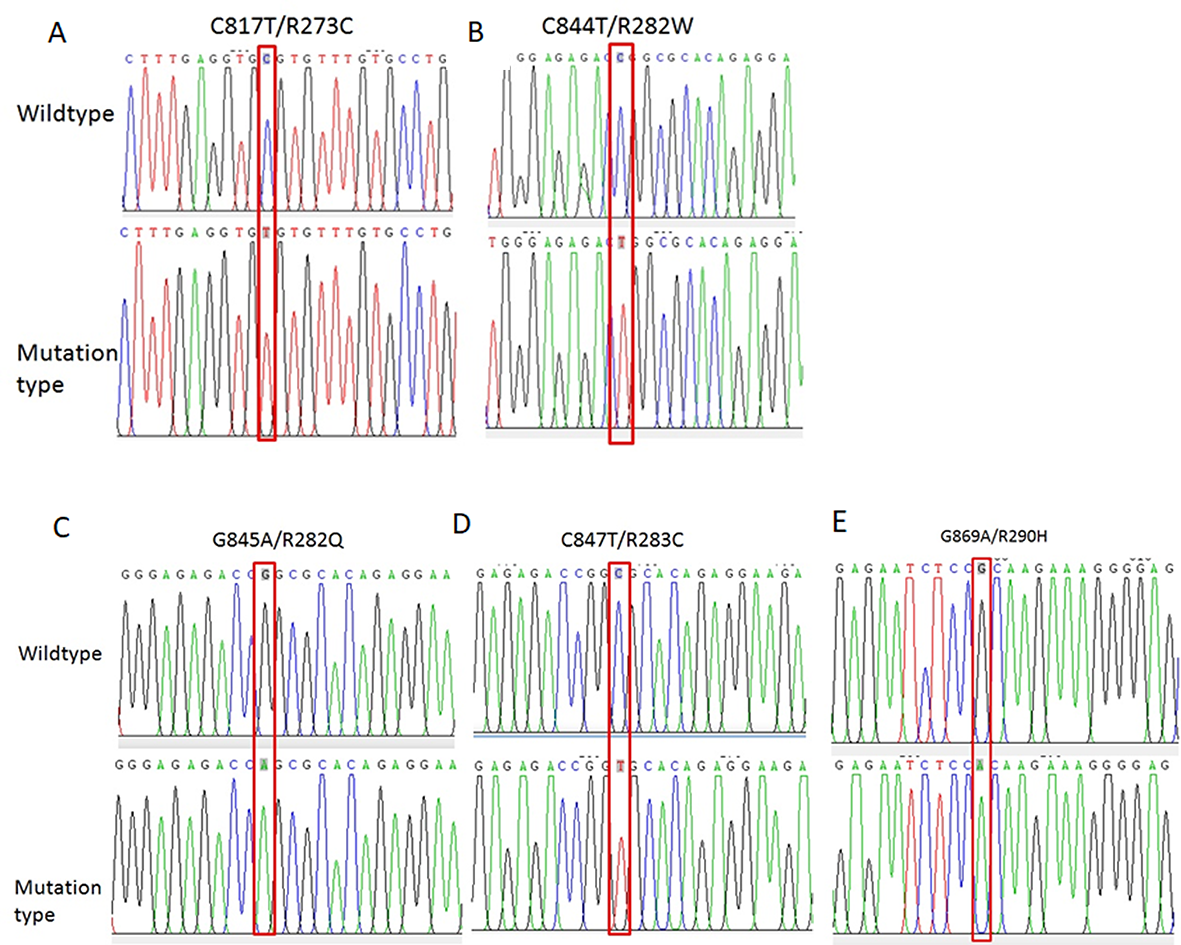

Supplement: Supplementary file 1 [file DataSheet_1.zip › supplementary/Figure S1.tif]

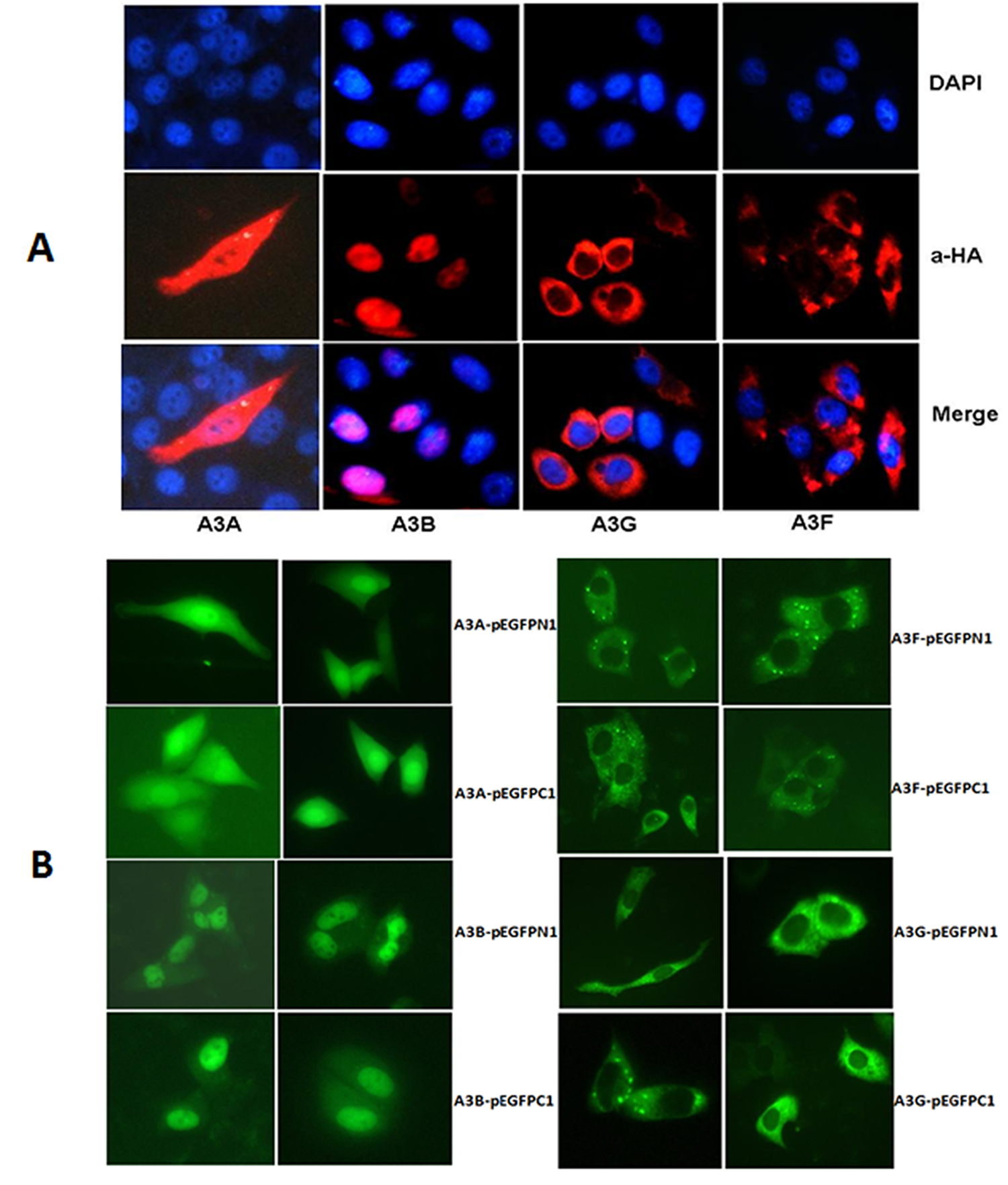

Supplement: Supplementary file 1 [file DataSheet_1.zip › supplementary/Figure S2.tif]

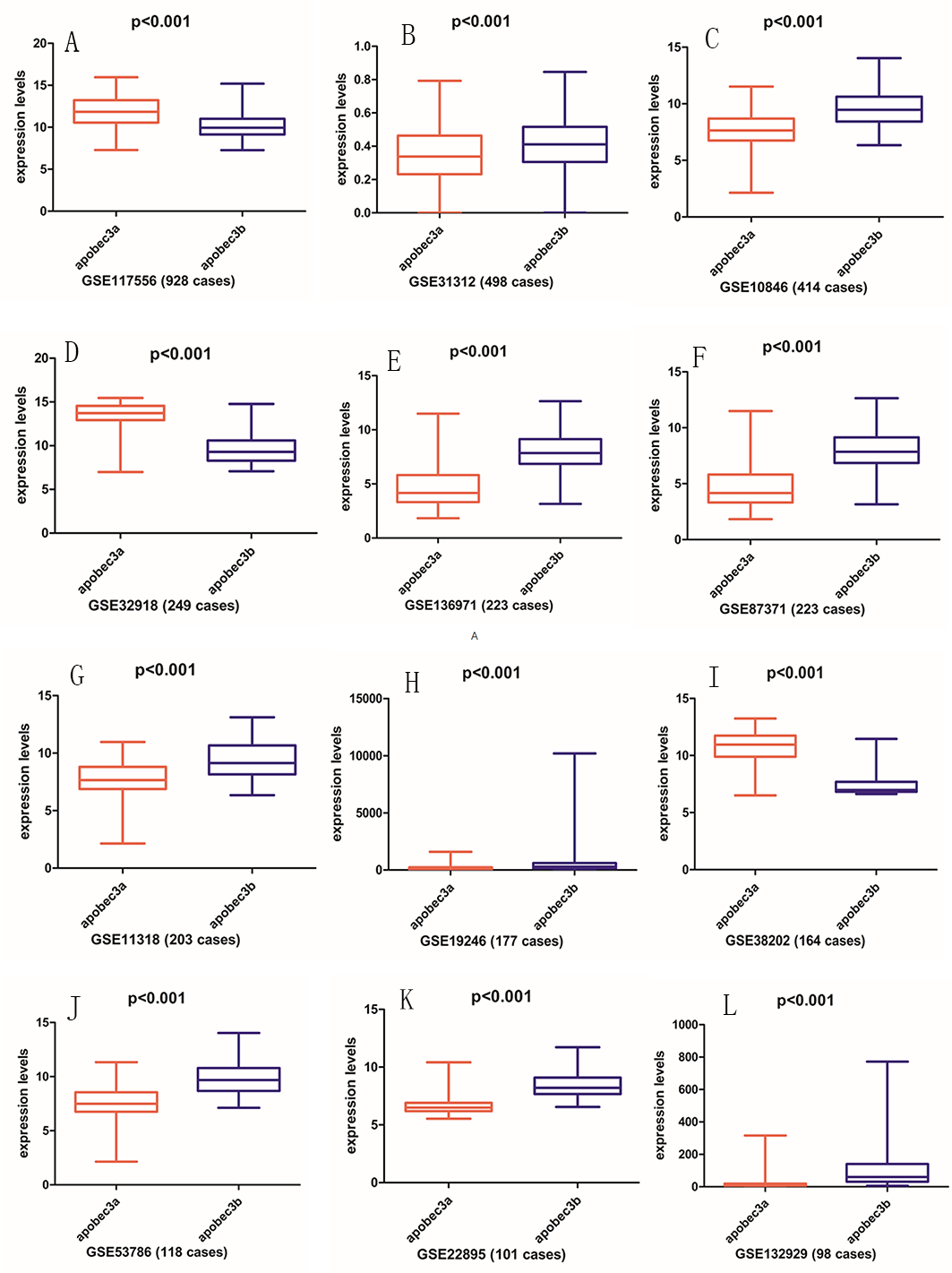

Supplement: Supplementary file 1 [file DataSheet_1.zip › supplementary/Figure S3-GEO database.tif]

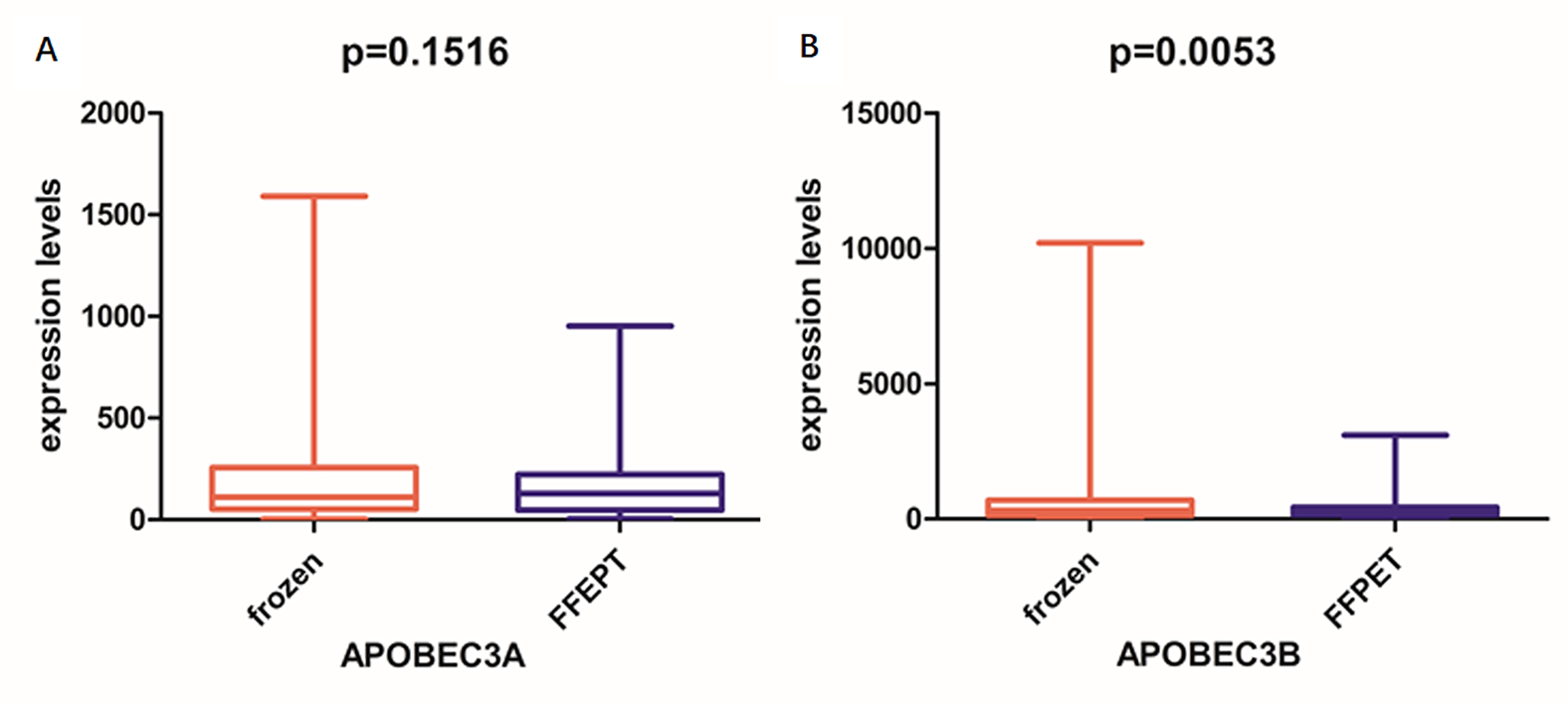

Supplement: Supplementary file 1 [file DataSheet_1.zip › supplementary/Figure S4-A3A and A3B in frozen vs FFPET.tif]

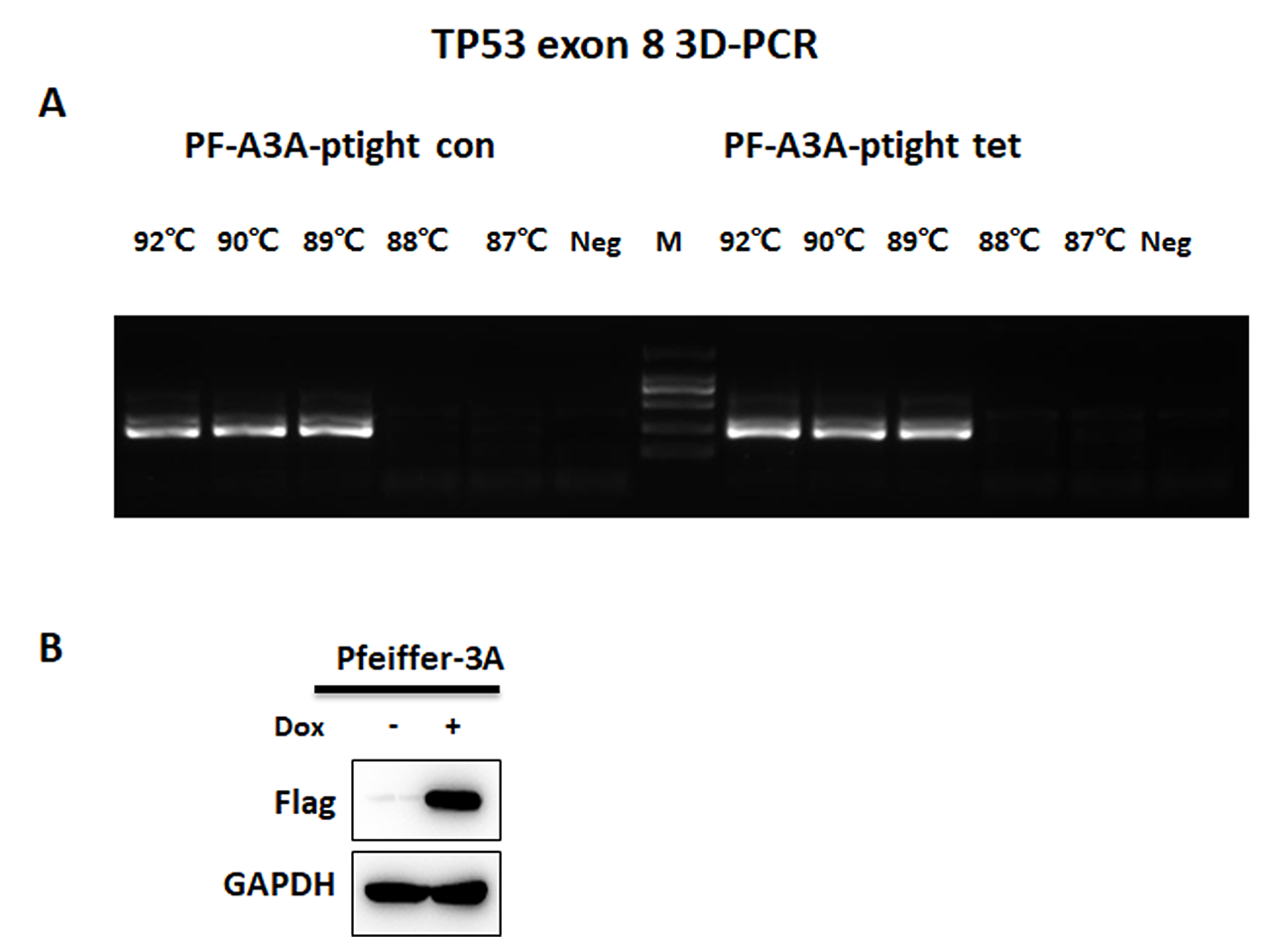

Supplement: Supplementary file 1 [file DataSheet_1.zip › supplementary/Figure S5.tif]
